# Supplementary figures and images for: Peripheral nervous system involvement associated with COVID-19. A systematic review of literature
Source: PLoS One. 2023 Apr 6;18(4):e0283827. doi: 10.1371/journal.pone.0283827 (PMC10079054; doi:10.1371/journal.pone.0283827)

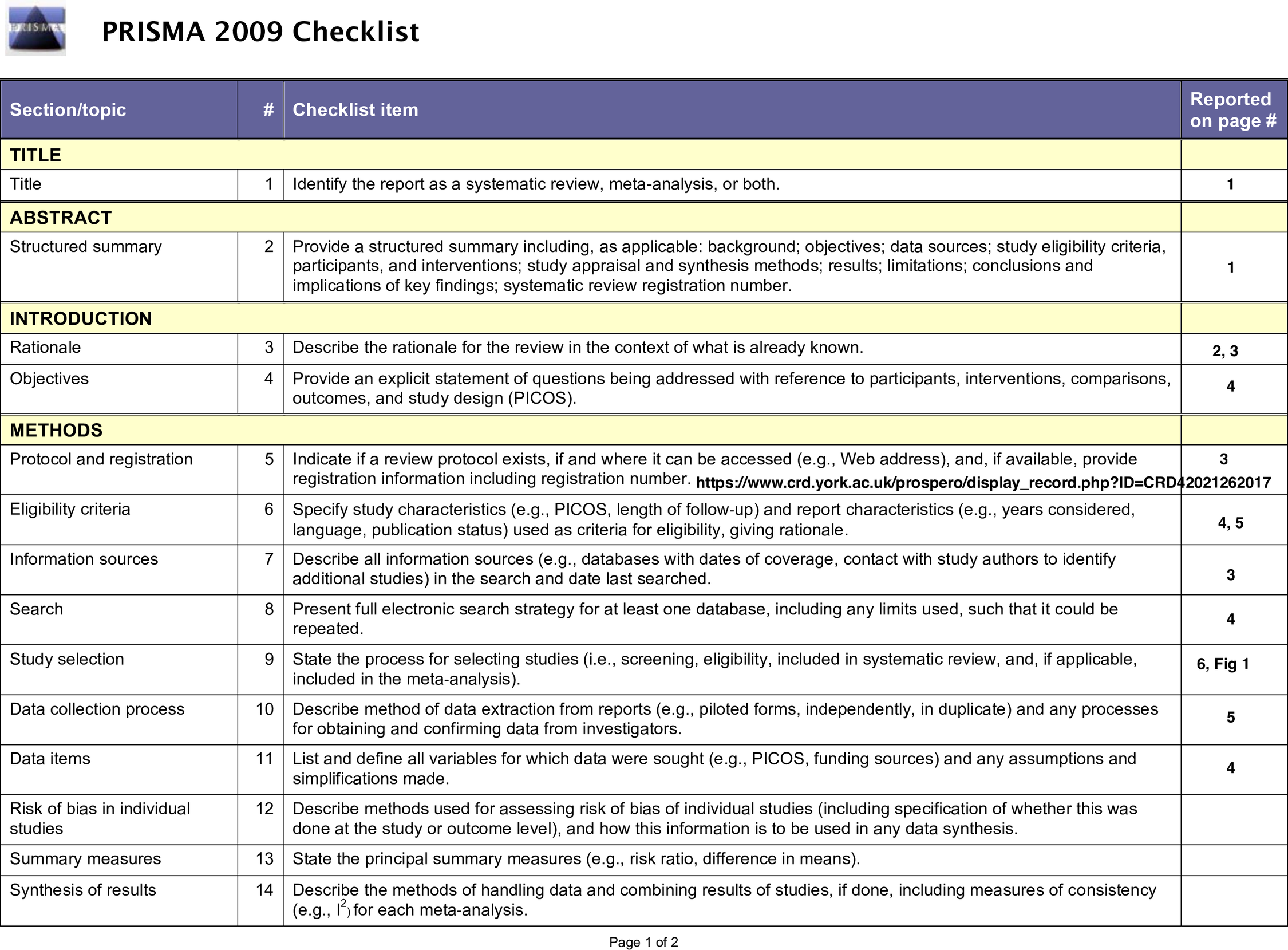

Supplement: S1 Fig — (TIF) [file pone.0283827.s001.tif]
