# Supplementary material for: Peripheral nervous system involvement associated with COVID-19. A systematic review of literature
Source: PLoS One. 2023 Apr 6;18(4):e0283827. doi: 10.1371/journal.pone.0283827 (PMC10079054; doi:10.1371/journal.pone.0283827)
Supplement: S1 Table — (DOCX) [file pone.0283827.s002.docx]

**Supplement Table 1. Characteristics of patients included in the qualitative analysis**

| No. | Author | No of patients | sex | COVID disease type | PNS | CN | Diagnosis | Neurological treatment | Neurological outcome |
| --- | --- | --- | --- | --- | --- | --- | --- | --- | --- |
|  | Jeong M. et al. [1] | 10 | 4F:6M | 2 severe | - | 10 VIII | Audiometry, MRI | GC | 2 - complete recovery  5 – no improvement  3 – mild improvement |
|  | Chowdury F. et. al. [2] | 1 | M | Non severe | GBS | - | EMG, CSF | IVIG | Mild/moderate sequelae |
|  | Roncati L. et. al. [3] | 2 | 2 M | Non-severe | carpal tunnel | - | EMG | surgical decompresion | Mild/moderate sequelae |
|  | Maramattom et.al. [4] | 1 | M | NA | GBS | - | EMG, CSF | IVIG | Mild/moderate sequelae |
|  | Mehta S. et. al. [5] | 1 | M | Non severe | GBS | - | EMG | IVIG | Mild/moderate sequelae |
|  | Defabio A. et. al. [6] | 1 | F | NA | GBS | - | CSF | IVIG | Mild/moderate sequelae |
|  | Masuccio F. et. al. [7] | 1 | F | NA | AMAN | - | EMG, CSF | PLEX +IVIG | Mild/moderate sequelae |
|  | García-Romo E. et. al. [8] | 1 | F | Non severe | - | VIII | MRI | NA | Mild/moderate sequelae |
|  | Chirakkal P. et. al. [9] | 1 | F | Non severe | - | VIII | audiometry | NA | Mild/moderate sequelae |
|  | Pelea T et. al. [10] | 1 | F | Non severe | AMAN | - | EMG, CSF, MRI | PLEX +IVIG | Mild/moderate sequelae |
|  | Hanif M. et. al. [11] | 1 | F | Non severe | AIDP | - | EMG, CSF | PLEX | Mild/moderate sequelae |
|  | Ricciardiello F. et. al. [12] | 5 | 2F:3M | Non severe |  | VIII | audiometry | GC | Mild/moderate sequelae |
|  | Hasibi M. et. el. [13] | 1 | M | Non severe |  | VII | clinical | GC | Mild/moderate sequelae |
|  | D'Orsi G. et. al. [14] | 1 | M | Non severe | AMSAN | - |  | PLEX, IVIG | Mild/moderate sequelae |
|  | Khan F. et. al. [15] | 5 | 3F:2M | Non severe | 3 AIDP  1 AMAN  1AMSAN | - | EMG, CSF | 4 IVIG  1 no treatement | 4 Mild/moderate sequelae  1 Death |
|  | Edwards M et. al. [16] | 1 | F | Non severe | SSNHL | VIII | audiometry | GC | Mild/moderate sequelae |
|  | Viatge T. et. al. [17] | 1 | M | Severe | Parsonage-Turner | - | EMG | kinetotherapy | Mild/moderate sequelae |
|  | Asfour L. et. al. [18] | 1 | M | Severe | SSNHL | VIII | audiometry | cochlear implant | Mild/moderate sequelae |
|  | Deane K. el.al. [19] | 1 | M | 4 | ON | II | MRI | GC | Mild/moderate sequelae |
|  | Duran TI. et. al. [20] | 1 | M | 3 | SSNHL | VIII | Clinical | GC, cyclophosphamide | No improvement |
|  | Manganotti P. et al. [21] | 3 | 3M | severe | - | 3 VII | CSF | IVIG | Mild/moderate sequelae |
|  | Mehrpour M. et. al. [22] | 1 | F | Non severe | AIDP | - | EMG | IVIG | Mild/moderate sequelae |
|  | Acharya S. et. al. [23] | 1 | M | Severe | MNM | - | EMG, MRI | kinetotherapy | Mild/moderate sequelae |
|  | Koca A. et.al. [24] | 1 | M | Non severe | AIDP | - | EMG | PLEX, IVIG | Mild/moderate sequelae |
|  | Babu TA. et. al. [25] | 1 | M | Non severe |  | VIII | Clinical | GC | Mild/moderate sequelae |
|  | Nejad H. et. al. [26] | 1 | M | Severe | GBS | - | CSF | IVIG | Death |
|  | Fletman EW. et. al. [27] | 1 | F | Severe | AMSAN | - | CSF, EMG | IVIG | Mild/moderate sequelae |
|  | Gerstacker K. et.al. [28] | 1 | M | Severe | SSNHL | VIII |  | GC | No improvement |
|  | Chen T. et.al. [29] | 1 | F | Non severe | GBS | - | MRI, CSF, EMG | NA | NA |
|  | Garcia J. et. al. [30] | 1 | F | Non severe | AIDP | VII, IX, X | EMG, CSF | IVIG | Mild/moderate sequelae |
|  | Abdelnasser A. et. al. [31] | 1 | F | Non severe | AIDP | - | EMG, CSF | GC | NA |
|  | Daia C. et. al. [32] | 10 | 6F:2M | NA | MNM | - | EMG | NA | Mild/moderate sequelae |
|  | Rahimi V. et. al. [33] | 1 | F | Non severe | SSNHL | VIII | MRI, audiometry | GC | Mild/moderate sequelae |
|  | Papri N. et. al. [34] | 1 | M | Non severe | AIDP | - | EMG | IVIG | Mild/moderate sequelae |
|  | Gagarkin DA. et. al. [35] | 1 | F | Non severe | GBS | IX, X | MRI | IVIg | Mild/moderate sequelae |
|  | Pujol Lucas C. et. al. [36] | 1 | F | Non severe | AIDP | - | EMG | IVIG | Mild/moderate sequelae |
|  | Coll C. et. al. [37] | 2 | 2 M | Severe |  | 2 XI | EMG | NA | Mild/moderate sequelae |
|  | Yiu CA. et. al. [38] | 1 | M | Non severe | AIDP | - | EMG, CSF | IVIG | Mild/moderate sequelae |
|  | Anilkumar A. et. al. [39] | 1 | F | Non severe |  | VI | MRI, CSF | No treatment | Mild/moderate sequelae |
|  | Ates MF. et. al. [40] | 1 | F | Non severe |  | III | CSF, CT | GC | Mild/moderate sequelae |
|  | Kerstens J. et. al. [41] | 1 | M | Non severe |  | VII | MRI | GC | Mild/moderate sequelae |
|  | Douedi S. et. al. [42] | 1 | M | Non severe |  | I, III, VII | MRI | No treatment | Mild/moderate sequelae |
|  | Dias F. et. al. [43] | 1 | M | Severe | radiculitis | - | MRI, CSF, EMG | IVIG | Mild/moderate sequelae |
|  | Ebrahimzadeh SA. et. al. [44] | 2 | 2 M | Non severe | 2 AIDP | 1 VII | EMG, CSF | No treatment | Mild/moderate sequelae |
|  | Sharma A. et.al. [45] | 1 | F | Non severe |  | II | OCT, MRI | GC | Mild/moderate sequelae |
|  | Brown M. et. al. [46] | 1 | M | Non severe | GBS | VII | MRI, CSF | IVIG, PLEX | Mild/moderate sequelae |
|  | Mokhashi N. et. al. [47] | 1 | F | Non severe | GBS | - | CSF | IVIG | Mild/moderate sequelae |
|  | Yakobi J. et.al. [48] | 1 | M | Non severe | GBS | - | CSF | IVIG | Mild/moderate sequelae |
|  | Han C. et. el. [49] | 1 | M | Severe | brachial plexopathy | - | MRI, EMG | physiotherapy | Mild/moderate sequelae |
|  | Mackenzie N. et al. [50] | 1 | F | Non severe | GBS | VII | CSF, EMG | PLEX | Mild/moderate sequelae |
|  | Rodriguez- Rodriguez M. et. al. [51] | 1 | F | Non severe | ON | II | OCT | GC | No improvement |
|  | Ismail I. et. al. [52] | 1 | M | Non severe | Parsonage Turner | - | MRI, EMG | GC,IVIG | No improvement |
|  | Svačina M. et. al. [53] | 3 | 1F:2M | Non severe | 2 AIDP+AMSAN  1 AIDP+CN | III, VII,IX, X  XII | EMG, CSF | 3 IVIG  1 GC | No improvement |
|  | Amanat M. et. al. [54] | 1 | F | Non severe | AMAN | VII | EMG, CSF | IVIG | Mild/moderate sequelae |
|  | Oke IO. et.al. [55] | 1 | M | Non severe |  | VII | MRI | GC | Mild/moderate sequelae |
|  | de Franciso Moure J et.al. [56] | 5 | 3F:2M | Non severe | GBS | - | EMG | No treatment | 3 Mild/moderate sequelae  2 Complete recovery |
|  | Beckers E. et. al. [57] | 1 | M | Non severe | SSNHL | VIII | audiometry | prednisone, ITS | No improvement |
|  | Singh R. et.al. [58] | 1 | M | Non severe | AMSAN | VII | CSF, EMG | IVIG | Mild/moderate sequelae |
|  | Molina Gil J. et.al. [59] | 1 | M | Non severe |  | V | MRI | No treatment | Complete recovery |
|  | Kumar V. et.al. [60] | 1 | F | Non severe |  | VII | clinical | GC | Complete recovery |
|  | Todisco M. et.al. [61] | 4 | 3M:1F | Severe | - | X, XII, XII | MRI, EMG, CSF | NA | No improvement |
|  | Tutar K. et.al. [62] | 1 | M | NA |  | 1 III | 1 MRI | dilute pilocarpin 0,1% | No improvement |
|  | Bartoli A. et.al. [63] | 1 | F | Severe | AMAN | - | EMG | IVIG | Mild/moderate sequelae |
|  | Neo WL. et. al. [64] | 2 | 2 M | Non severe |  | VII | MRI | GC | 1 Mild/moderate sequelae  1 complete recovery |
|  | Börü UT. et. al. [65] | 1 | M | Non severe | GBS | - | EMG | No treatment | Complete recovery |
|  | Meshref M. et.al. [66] | 1 | F | Non severe | GBS | VII, IX, X | MRI | IVIG | Mild/moderate sequelae |
|  | Tekin AB. et. al. [67] | 1 | M | Non severe | AMSAN | - | EMG, CSF | IVIG | Mild/moderate sequelae |
|  | Rodrigo Armenteros P. et. al. [68] | 1 | M | Severe |  | II | MRI, CSF | NA | Mild/moderate sequelae |
|  | Sabharwal P. et.al. [69] | 1 | M | Severe | AMSAN | - | MRI, CSF, EMG | IVIG | Mild/moderate sequelae |
|  | Goel K. el.al. [70] | 1 | 2 | Non severe | GBS | - | MRI, CSF, EMG | IVIG | NA |
|  | Francois J. et.al.[71] | 1 | F | NA | ON | II | MRI, CSF | GC | No improvement |
|  | Bahouth S. et.al. [72] | 1 | M | Severe | AMSAN | - | MRI, EMG | NA | No improvement |
|  | De Gennaro R. et. al. [73] | 2 | 2M | Severe |  | 2XII, 1CBH | 1 EMG, 2 CSF | IVIG | 1 Mild/moderate sequelae  1 complete recovery |
|  | Raahimi M. et. al. [74] | 1 | M | Severe | AIDP | - | EMG, CSF, MRI | IVIG | Complete recovery |
|  | Zubair A. et. al. [75] | 2 | 2M | Severe | AMSAN | - | EMG, CSF | IVIG | Mild/moderate sequelae |
|  | Dufour C. et.al. [76] | 1 | F | Non severe | GBS | - | Anti GM1 | IVIG | Mild/moderate sequelae |
|  | Dahl EH. et.al. [77] | 1 | M | Non severe |  | VII | anti-GD1b IgG, anti-GM1 IgG, CSF | NA | Complete recovery |
|  | Francis JE [78] | 1 | F | Non severe |  | I, VI | Prism cover test | NA | Complete recovery |
|  | Khedr E. et.al.[79] | 5 | 3M:2F | severe | AIDP | 1 VI, 1VII | EMG | PLEX, IVIG, CS | 3 complete recovery  2 Mild/moderate sequelae |
|  | Singhai A. et.al. [80] | 1 | M | severe | AIDP | - | EMG | IVIG | Complete recovery |
|  | Zoric L. et.al. [81] | 1 | M | Non severe |  | II | OCT, anti MOG | GC | Complete recovery |
|  | Halalau A. et.al. [82] | 1 | M | Non severe |  | VIII | Angio CT | GC | Complete recovery |
|  | Paradis J. et.al. [83] | 1 | F | severe | AIDP | IX, X | EMG, CSF | IVIG | Mild/moderate sequelae |
|  | Ansari B. et.al. [84] | 1 | F | severe | AIDP | - | MRI, EMG | PLEX | Mild/moderate sequelae |
|  | Bastola A. et.al. [85] | 1 | M | Non severe |  | VII | MRI | GC | Complete recovery |
|  | Manganotti P. et.al. [86] | 5 | 1F:4M | 3 severe:2 non severe | GBS | III, V, VII | MRI, CSF, EMG | 4 IVIG, 1GC | 4 Mild/moderate sequelae  1 no improvement |
|  | Agosti E. et.al. [87] | 1 | M | Non severe | GBS | VII | EMG | IVIG 5 days | Mild/moderate sequelae |
|  | Li CW. et. al.[88] | 1 | M | Non severe |  | I | MRI | No treatment | Mild/moderate sequelae |
|  | Aasfara J. et. al. [89] | 1 | F | Non severe | GBS: | VIIIVII | MRI, EMG, CSF | IVIG, GC | Mild/moderate sequelae |
|  | Colonna S. et. al. [90] | 1 | M | Severe | GBS | - | MRI, EMG, CSF | IVIG | Mild/moderate sequelae |
|  | Eslamian F. et. al. [91] | 1 | M | Severe | AMAN | - | EMG | IVIG | Mild/moderate sequelae |
|  | Kumar H. et. al. [92] | 2 | 2M | Severe | 2 AMAN + AIDP | - | EMG | IVIG | 1 Complete recovery  1 Mild/moderate sequelae |
|  | Moyano AJ. et.al. [93] | 1 | F | Non severe |  | VII, IX, X | Swallowing study | GC | Mild/moderate sequelae |
|  | Sisniega D. [94] | 1 | F | Severe | AIDP | - | CSF | PLEX | Mild/moderate sequelae |
|  | Bueso T. et. al. [95] | 1 | F | Severe |  | - | clinical | IVIG | Mild/moderate sequelae |
|  | Abolmaali M. et. al. [96] | 3 | 1F:2M | Severe | 2 AMSAN  1 GBS | - | EMG, CSF | 3 PLEX , 1 IVIG | 1 Mild/moderate sequelae  2 death |
|  | Karimi-Galougahi M. et. al. [97] | 1 | M | Non severe |  | VII | EMG, PET CT | NA | NA |
|  | Nanda S. et. al. [98] | 1 | F | Non severe | GBS | - | EMG, CSF | IVIG | Mild/moderate sequelae |
|  | Gogia B. et. al. [99] | 1 | M | Non severe |  | V, VII | MRI, CSF | GC | Mild/moderate sequelae |
|  | Soliman S. et.al. [100] | 2 | 2 M | Severe | 2 AMSAN | - | EMG, CSF | NA | NA |
|  | Judge C. et. al. [101] | 1 | M | Non severe |  | VII | MRI, CSF | No treatment | Mild/moderate sequelae |
|  | Petrelli C. et. al. [102] | 1 | M | Non severe | AMAN | VII | EMG | IVIG, GC | Mild/moderate sequelae |
|  | Raharimanantsoa O. et. al. [103] | 1 | F | Non severe |  | I, III | clinical | GC | Mild/moderate sequelae |
|  | Ghosh R. et. al. [104] | 1 | F | Non severe | dysautonomia; AMAN | - | EMG, MRI | IVIG | Mild/moderate sequelae |
|  | Cabañes-Martínez L. et. al. [105] | 4 | 4M | Severe | 4 AMSAN | - | EMG | 3GC, 1 no treatment | NA |
|  | Lima M. et. al. [106] | 8 | 7F:1M | Non severe | F | VII | MRI, CSF | GC | Mild/moderate sequelae |
|  | Costa Martins D. et. al. [107] | 1 | M | Severe |  | XII | clinical | neuromotor rehabilitation | Mild/moderate sequelae |
|  | Greer C. [108] | 3 | 1F:2M | Non severe | 1 AMAN | 2 VI | 2 clinical,  1 EMG | 2 No treatment  1 IVIG | 1 Mild/moderate sequelae  2 complete recovery  3 no improvement |
|  | Ordás C. et. al. [109] | 1 | M | Non severe | GBS | VI | EMG | GC | complete recovery |
|  | Civardi C. et. al. [110] | 1 | M | Severe | GBS | VII | EMG | IVIG | Mild/moderate sequelae |
|  | Derollez C. et. al. [111] | 1 | F | Severe |  | VII | CSF, EMG | No treatment | Complete recovery |
|  | Virgo J. et. al. [112] | 1 | M | Non severe | AMAN |  |  | NA | NA |
|  | Karimi-Galougahi M. et. al. [113] | 3 | 2F:1M | Non severe |  | VIII | audiometry | NA | NA |
|  | Gale A. et. al. [114] | 1 | M | Severe | GBS | VII | EMG | IVIG | NA |
|  | Vanaparthy R. et. al. [115] | 1 | F | Non severe |  | VIII | clinical | GC | NA |
|  | Kushwaha S. et. al. [116] | 1 | M | Non severe | GBS | - | EMG | IVIG | NA |
|  | Liberatore G. et. al. [117] | 1 | M | Severe | PCB GBS | IX, X, VII, XII | EMG, CSF, MRI | No treatment | Complete recovery |
|  | McDonnell E. et. al. [118] | 1 | M | Non severe | GBS | VII | EMG, CSF | IVIG | Complete recovery |
|  | Kanjwal K. et. al. [119] | 1 | F | Non severe | disautonomy | - | Tilt-test | No treatment | Mild/moderate sequelae |
|  | Bureau B. et. al. [120] | 1 | F | Non severe | PNP | - | MRI, CSF | GC, IVIG | Complete recovery |
|  | Othenin-Girard A. et. al. [121] | 1 | M | Non severe | MNM |  | MRI, CSF, EMG | IVIG, GC, cyclophosphamide, rytuximab | Complete recovery |
|  | Lowery M. et. al. [122] | 1 | M | Severe | MFS overlap GBS | III, IV, VI | CSF, MRI | IVIG | Mild/moderate sequelae |
|  | Boostani R. et. al. (123) | 1 | M | Severe | AIDP | - | EMG | IVIG | No improvement |
|  | Groiss S. et. al. [124] | 1 | F | Severe | GBS | VII | EMG, CSF | NA | NA |
|  | Senel M. et. al. [125] | 1 | M | Non severe | MFS | III, IV,VI | EMG, CSF | IVIG | complete recovery |
|  | Bracaglia M. et. al. [126] | 1 | F | Severe | AIDP | VII | EMG, CSF | IVIG | Mild/moderate sequelae |
|  | Faucher A. et. al. [127] | 1 | M | Non severe |  | III | MRI, CSF | No treatment | Complete recovery |
|  | Esteban Molina A. et. al. [128] | 1 | F | Non severe | GBS | VII, IX, X | MRI, EMG, CSF | IVIG | slight improvement |
|  | Chan J. et. al. [129] | 1 | M | Non severe | GBS | VII | EMG | IVIG | Mild/moderate sequelae |
|  | Khaja M. et. al. [130] | 1 | M | Non severe | GBS, Bell's palsy | VII | MRI, CSF | IVIG | Mild/moderate sequelae |
|  | Hirayama T. et. al. [131] | 1 | F | Non severe | GBS | - | EMG, CSF | no treatment | Mild/moderate sequelae |
|  | García-Manzanedo S. et. al. [132] | 1 | M | Non severe | GBS | VII , IX , X | EMG, CSF | IVIG | Mild/moderate sequelae |
|  | Belghmaidi S. et. al. [133] | 1 | F | Non severe |  | III, IV, VI | clinical | Non treatment | Complete recovery |
|  | Faqihi F. et.al. [134] | 1 | M | Severe | PNP | - | EMG | PLEX | Complete recovery |
|  | Ribeiro B. et. al. [135] | 1 | M | NA |  | VII | MRI | NA | NA |
|  | Agha Abbaslou M. et. al. [136] | 1 | F | Severe | AMSAN | - | EMG, CSF | IVIG | no improvement |
|  | Lang B. et. al. [137] | 1 | F | Non severe |  | VIII | MRI, audiometry | GC | no improvement |
|  | Redondo-Urda M. et. al. [138] | 1 | F | Non severe | GBS | - | EMG, CSF | IVIG | Mild/moderate sequelae |
|  | Diez-Porras L. et. al. [139] | 1 | M | Severe | GBS | - | EMG, CSF | IVIG | Mild/moderate sequelae |
|  | Abrams R. et. al. [140] | 1 | F | Severe | GBS | - | CSF | PLEX | Mild/moderate sequelae |
|  | Siepmann T. et.al. [141] | 1 | M | Non severe | MNM | - | EMG | GC | no improvement |
|  | Korem S. et. al. [142] | 1 | F | Non severe | GBS | - | CSF | IVIG | Mild/moderate sequelae |
|  | Ameer N. et. al. [143] | 1 | M | Non severe | GBS | - | EMG | IVIG | Mild/moderate sequelae |
|  | Granger A. et al. [144] | 1 | M | Non severe | GBS | - | EMG | PLEX 5 sessions | Mild/moderate sequelae |
|  | Wada S. et. al. [145] | 1 | M | Severe | GBS | - | EMG | IVIG | Mild/moderate sequelae |
|  | Pascual-Goñi E. et. al. [146] | 1 | F | Severe |  | VI | MRI | none | no improvement |
|  | Hutchins K. et. al. [147] | 1 | M | severe | GBS | VII, V | MRI, CSF | PLEX | Mild/moderate sequelae |
|  | Kilinc D. et. al. [148] | 1 | M | Non severe | GBS | VII | EMG | IVIG | Mild/moderate sequelae |
|  | Oguz-Akarsu E. et. al. [149] | 1 | F | Non severe | 3 GBS | - | EMG | PLEX | Mild/moderate sequelae |
|  | Lascano A. et. al. [150] | 3 | 3F | 1 non severe, 2 severe | GBS | - | EMG | IVIG | Mild/moderate sequelae |
|  | Bigaut K. et. al. [151] | 2 | 1F:1M | Non severe | 2 GBS | 2 VII | EMG | IVIG | Mild/moderate sequelae |
|  | Fernández-Domínguez J. et. al. [152] | 1 | F | Non severe | GBS | - | EMG | IVIG | Mild/moderate sequelae |
|  | Atakla H. et. al. [153] | 1 | M | severe | AIDP | - | EMG | IVIG | Mild/moderate sequelae |
|  | Goh Y. et. al. [154] | 1 | M | Non severe |  | VII |  | GC | no improvement |
|  | Ray A. [155] | 1 | M | Non severe | MFS |  | clinical | No treatment | Mild/moderate sequelae |
|  | Figueiredo R. et. al. [156] | 1 | F | Non severe |  | VII | clinical | GC | Mild/moderate sequelae |
|  | Dinkin M. et.al. [157] | 2 | 2M | Non severe | 1 MFS | 2 VI | MRI | IVIG | Mild/moderate sequelae |
|  | Gutiérrez-Ortiz C. et. Al. [158] | 2 | 2M | Non severe | MFS | III, IV, VI | clinical | IVIG | Complete recovery |
|  | Naddaf E. et. al. [159] | 1 | F | Non severe | GBS | - | EMG | PLEX | Mild/moderate sequelae |
|  | Falcone M. et. al. [160] | 1 | M | Severe |  | VI | Clinical | No treatment | no improvement |
|  | Kilic O. [161] | 1 | M | Non severe |  | VIII | Audiometry | No treatment | complete recovery |
|  | Aoyagi Y. et. al. [162] | 1 | M | Severe |  | IX, X | Clinical | No treatment | Mild/moderate sequelae |
|  | Manganotti P. et al. [163] | 1 | F | Severe | MFS | III, V, VII | Clinical | IVIG | Mild/moderate sequelae |
|  | Su X. et. al. [164] | 1 | M | Severe | GBS | - | Clinical | IVIG | Mild/moderate sequelae |
|  | Tiet M. et. al. [165] | 1 | M | Non severe | GBS | VII | EMG | IVIG | Mild/moderate sequelae |
|  | Paybast S. et. al. [166] | 1 | M | Non severe | GBS, | VII | EMG | PLEX | Mild/moderate sequelae |
|  | Casas E. et. al. [167] | 1 | M | Non severe |  | VII | MRI | No treatment | complete recovery |
|  | Guijarro-Castro C. et. al. [168] | 1 | M | Severe | GBS | - | EMG | IVIG | Mild/moderate sequelae |
|  | Sancho-Saldaña A. et. al. [169] | 1 | F | Non severe | GBS | VII, IX, X | EMG | IVIG | No improvement |
|  | Assini A. et. al. [170] | 2 | 2M | Severe | 1 GBS + MFS  1 GBS | III, IX, X | EMG | IVIG | 1 Mild/moderate sequelae  1 Complete recovery |
|  | Lantos J et. al. [171] | 1 | M | Non severe | GBS MFS | III | MRI | IVIG | Mild/moderate sequelae |
|  | Arnaud S. et. al. [172] | 1 | M | Non severe | GBS | - | EMG | IVIG | Mild/moderate sequelae |
|  | Juliao Caamaño D. et. al. [173] | 1 | M | Non severe |  | VII | clinical | GC | Mild/moderate sequelae |
|  | Pfefferkorn T. et. al. [174] | 1 | M | Severe | GBS | - | EMG | IVIG, PLEX | No improvement |
|  | Coen M. et. al. [175] | 1 | M | Non severe | GBS dysautonomia | - | clinical | IVIG | Complete recovery |
|  | Alberti P. et. al. [176] | 1 | M | Severe | GBS | - | EMG | IVIG | Death |
|  | Padroni M. et. al. [177] | 1 | F | Non severe | GBS | - | EMG | IVIG | NA |
|  | Malayala S. et. al. [178] | 1 | F | Non severe |  | VIII |  | No treatment | Complete recovery |
|  | Toscano G. [179] | 4 | 1F:3M | 2 Severe, 2 non severe | 4 GBS | 1 IX, X  1 VII |  | IVIG | 3 Mild/moderate sequelae  1 no improvement |
|  | Farzi M. et. al. [180] | 1 | M | NA | AIDP | - | EMG | IVIG | Mild/moderate sequelae |
|  | Webb S. et. al. [181] | 1 | M | NA | AIDP | - | EMG | IVIG | Mild/moderate sequelae |
|  | Rana S. et. al. [182] | 1 | M | NA | AIDP and MFS | III, IV, VI, VII | EMG | IVIG | NA |
|  | Ottaviani D. et. al. [183] | 1 | F | Severe | GBS | - | EMG | IVIG | no improvement |
|  | Scheidl E. et. al. [184] | 1 | F | NA | AIDP | - | EMG | IVIG | Mild/moderate sequelae |
|  | Otmani EI. et. al. [185] | 1 | F | Non severe | AMSAN | - | EMG | IVIG | no improvement |
|  | Abdelnour L. et. al. [186] | 1 | F | Non severe | PNP | - | clinical | No treatment | complete recovery |
|  | Camdessanche J. et. al. [187] | 1 | F | NA | AIDP | - | EMG | IVIG | NA |
|  | Sedaghat Z. et. al. [188] | 1 | M | Non severe | AMAN | VII | EMG | IVIG | NA |
|  | Maurier F. et. al. [189] | 1 | F | Non severe |  | X | clinical | NA | NA |
|  | Ghiasvand F. et. al. [190] | 1 | F | severe | GBS | - | EMG | GC | Death |
|  | Marta-Enguita J. et. al. [191] | 1 | F | Non severe | GBS | IX, X | clinical | NA | Death |
|  | Velayos Galán A. et.al. [192] | 1 | M | severe | GBS | VII, IX, X | EMG | IVIG | Mild/moderate sequelae |
|  | Zhao H. et. al. [193] | 1 | F | Non severe | GBS | - | EMG | IVIG | complete recovery |
|  | Wei H. et. al. [194] | 1 | M | Severe |  | III, IV, VI | clinical | IVIG, GC | Death |
|  | Virani A. et. al. [195] | 1 | M | Severe | GBS | - | clinical | IVIG | Mild/moderate sequelae |
|  | Rajdev K. et. al. [196] | 1 | M | Severe | GBS | - | EMG | IVIG, PEX | Complete recovery |
|  | Lampe A. et. al. [197] | 1 | M | Severe | GBS | - | EMG | IVIG | Mild/moderate sequelae |
|  | Homma Y. et. al. [198] | 1 | F | 2 Non severe  1 NA |  | I, VII | clinical | No treatment | complete recovery |
|  | Avenali M. et. al. [199] | 3 | 1F:2M | NA | 2 AIDP  1AMSAN | - | EMG, CSF | 3 IVIG  1PLEX | Mild/moderate sequelae |
|  | Egilmez O. et. al. [200] | 6 | 4F:2M | 1 severe  5 non severe |  | VII, I | MRI, CT | GC | 5 Mild/moderate sequelae  1 Exitus |
|  | Shouman K. et. al. [201] | 27 | 16F:11M | NA | dysautonomia | - | Autonomic tests | No treatment | NA |
|  | Bax F. et. al. [202] | 1 | M | Severe | AIDP | - | EMG, CSF | no treatment | Mild/moderate sequelae |
|  | Goodman B. et. al. [203] | 6 | 4F:2M | Non severe | dysautonomia | - | Autonomic tests | NA | NA |
|  | Guilmot A. et. al. [204] | 2 | NA | Non severe |  | V, VII, VIII  1 III | MRI, CSF | 1 GC  1 NA | 1 Mild/moderate sequelae  1 complete recovery |
|  | Klironomos S. et. al. [205] | 1 | F | NA | GBS | - | EMG, MRI, CSF | IVIG | Mild/moderate sequelae |
|  | Garnero M. et. al. [206] | 6 | 2F:4M | 3 severe  1 non severe | 3 AIDP  2 AMSAN  1 GBS+MFS | - | EMG, CSF | IVIG | 4 Mild/moderate sequelae  2 Complete recovery |

*Abbreviations: F – female, M – male, PNS – peripheral nervous system, CN – cranial nerves, CSF – cerebrospinal fluid, AIDP – acute inflammatory demyelinating polyneuropathy, AMAN – acute motor axonal neuropathy, AMSAN – acute motor and sensitive axonal neuropathy, GBS – Guillain Barre syndrome, MFS - Miller Fisher syndrome, PNP – polyneuropathy, MNM – mononeuritis multiplex, GC - glucocorticoids, PLEX – plasma exchange, IVIG – intravenous immunoglobulins, EMG – electromyography, FESC – feet electrochemical skin conductance, HESC – hand electrochemical skin conductance, I – olfactory nerve, II – optic nerve, III – oculomotor nerve, IV – trochlear nerve, V – trigeminal nerve, VI – abducens nerve, VII – facial nerve, VIII – vestibulocochlear nerve, IX – glossopharyngeal nerve, X – vagus nerve, XI – accessory nerve, XII – hypoglossal nerve, NA – not available*.

**References**

1. Jeong M, Ocwieja KE, Han D, Wackym PA, Zhang Y, Brown A, et al. Direct SARS-CoV-2 infection of the human inner ear may underlie COVID-19-associated audiovestibular dysfunction. Communications Medicine. 2021 Dec 29;1(1):44.

2. Chowdhury FUH, Paul S, Aman S, Haque A, Rafiquzzaman M, Rahman M, et al. A Middle-Aged Man Presented with Quadriparesis during COVID-19 Pandemic. Case Rep Neurol. 2021 Aug 18;13(2):529–34.

3. Roncati L, Gianotti G, Gravina D, Attolini G, Zanelli G, Rosa N della, et al. Carpal, cubital or tarsal tunnel syndrome after SARS-CoV-2 infection: A causal link? Med Hypotheses. 2021 Aug;153:110638.

4. Maramattom B, Zachariah A, Meleth H, Zachariah Z, Mathew K. Post Covid-19 guillain barre syndrome. Ann Indian Acad Neurol. 2021;0(0):0.

5. Mehta S, Sunder A. Getting paralysed after COVID: Guillain–Barre syndrome. J Family Med Prim Care. 2021;10(7):2706.

6. Defabio AC, Scott TR, Stenberg RT, Simon EL. Guillain-Barré syndrome in a patient previously diagnosed with COVID-19. Am J Emerg Med. 2021 Jul;45:154–5.

7. Masuccio FG, Barra M, Claudio G, Claudio S. A rare case of acute motor axonal neuropathy and myelitis related to SARS-CoV-2 infection. J Neurol. 2021 Jul 17;268(7):2327–30.

8. García-Romo E, Blanco R, Nicholls C, Hernández-Tejero A, Fernández-de-Arévalo B. COVID-19 asociada a nistagmo. Arch Soc Esp Oftalmol. 2021 Apr;96(4):224–6.

9. Chirakkal P, al Hail AN, Zada N, Vijayakumar DS. COVID-19 and Tinnitus. Ear Nose Throat J. 2021 Apr 4;100(2_suppl):160S-162S.

10. Pelea T, Reuter U, Schmidt C, Laubinger R, Siegmund R, Walther BW. SARS-CoV-2 associated Guillain–Barré syndrome. J Neurol. 2021 Apr 8;268(4):1191–4.

11. Hanif M. COVID-19-induced Guillain-Barre Syndrome: A Rare Complication of SARS-CoV-2 Infection. Journal of the College of Physicians and Surgeons Pakistan. 2021 Jul 1;31(2):S123–4.

12. Ricciardiello F, Pisani D, Viola P, Cristiano E, Scarpa A, Giannone A, et al. Sudden Sensorineural Hearing Loss in Mild COVID-19: Case Series and Analysis of the Literature. Audiol Res. 2021 Jul 1;11(3):313–26.

13. Hasibi M, Seyed Ahadi M, Abdollahi H, Jafari M. Protracted COVID-19 during Treatment of Facial Palsy. Case Rep Neurol Med. 2021 Jun 4;2021:1–3.

14. d’Orsi G, Sica S, Maiorano A, Melchionda D, Lalla A, Montemurro L, et al. Guillain-Barré syndrome as only manifestation of COVID-19 infection. Clin Neurol Neurosurg. 2021 Aug;207:106775.

15. Khan F, Sharma P, Pandey S, Sharma D, V. V, Kumar N, et al. COVID‐19‐associated Guillain‐Barre syndrome: Postinfectious alone or neuroinvasive too? J Med Virol. 2021 Oct 6;93(10):6045–9.

16. Edwards M, Muzaffar J, Naik P, Coulson C. Catastrophic bilateral sudden sensorineural hearing loss following COVID-19. BMJ Case Rep. 2021 Jun 24;14(6):e243157.

17. Viatgé T, Noel-Savina E, Prévot G, Faviez G, Plat G, de Boissezon X, et al. Syndrome de Parsonage-Turner compliquant une infection sévère à SARS-CoV-2. Rev Mal Respir. 2021 Oct;38(8):853–8.

18. Asfour L, Kay-Rivest E, Roland JT. Cochlear implantation for single-sided deafness after COVID-19 hospitalization. Cochlear Implants Int. 2021 Nov 2;22(6):353–7.

19. Deane K, Sarfraz A, Sarfraz Z, Valentine D, Idowu AR, Sanchez V. Unilateral Optic Neuritis Associated with SARS-CoV-2 Infection: A Rare Complication. American Journal of Case Reports. 2021 May 26;22.

20. Izci Duran T, Turkmen E, Dilek M, Sayarlioglu H, Arik N. ANCA-associated vasculitis after COVID-19. Rheumatol Int. 2021 Aug 7;41(8):1523–9.

21. Manganotti P, Pesavento V, Buoite Stella A, Bonzi L, Campagnolo E, Bellavita G, et al. Miller Fisher syndrome diagnosis and treatment in a patient with SARS-CoV-2. J Neurovirol. 2020 Aug 11;26(4):605–6.

22. Mehrpour M, Arab M, Hadavand F, Khalafi M, Khalafi M. A case report of Guillain–Barré syndrome in a pregnant woman infected by COVID-19. Acta Neurol Belg. 2021 Aug 3;121(4):1079–80.

23. Acharya S, Thibault M, Lee J, Taha O, Morpurgo AJ, Kshetree BK, et al. COVID-19-Induced Left Sciatic Neuropathy Requiring Prolonged Physical Medicine and Rehabilitation. Cureus. 2021 Jun 21;

24. Koca A, Talan L, Şahin T, Çınar G, Evren E, Karahan ZC, et al. An unusual course of SARS-CoV-2 infection: Challenging diagnosis of Guillain-Barré Syndrome. Tuberk Toraks. 2021 Jun 15;69(2):242–6.

25. Babu TA, Sarkar MK, Sharmila V. Covid-19 Vestibular Neuritis (Cvn) in a Healthcare Worker: A Rare Complication of Covid-19 Infection. Journal of the Royal College of Physicians of Edinburgh. 2021 Jun 1;51(2):200–200.

26. Nejad JH, Heiat M, Hosseini MJ, Allahyari F, Lashkari A, Torabi R, et al. Guillain-Barré syndrome associated with COVID-19: a case report study. J Neurovirol. 2021 Oct 27;27(5):802–5.

27. Fletman EW, Stumpf N, Kalimullah J, Levinson N, Deboo A. Guillain-Barré syndrome associated with COVID-19: an atypical, late-onset presentation. Neurological Sciences. 2021 Nov 22;42(11):4393–5.

28. Gerstacker K, Speck I, Riemann S, Aschendorff A, Knopf A, Arndt S. Deafness after COVID-19? HNO. 2021 Aug 21;69(S2):92–5.

29. Chen T, Briemberg HR. Guillain-Barre Syndrome with COVID-19. Canadian Journal of Neurological Sciences / Journal Canadien des Sciences Neurologiques. 2022 May 12;49(3):414–5.

30. Garcia JJ, Turalde CW, Bagnas MA, Anlacan VM. Intravenous immunoglobulin in COVID-19 associated Guillain–Barré syndrome in pregnancy. BMJ Case Rep. 2021 May 11;14(5):e242365.

31. Abdelnasser A, Mostafa M, Hasanin A, El-sakka A, Hassanein H. Guillain-Barré syndrome in the early post-partum period following COVID-19 infection. Int J Obstet Anesth. 2021 Aug;47:103172.

32. Daia C, Toader C, Scheau C, Onose G. Motor demyelinating tibial neuropathy in COVID-19. Journal of the Formosan Medical Association. 2021 Nov;120(11):2032–6.

33. Rahimi V, Asiyabar MK, Rouhbakhsh N. Sudden hearing loss and coronavirus disease 2019: the role of corticosteroid intra-tympanic injection in hearing improvement. J Laryngol Otol. 2021 May 29;135(5):464–6.

34. Papri N, Hayat S, Mohammed A, Afsar MdNA, Hasan I, Rahman A, et al. Guillain-Barré syndrome associated with SARS-CoV-2 infection: A case report with long term follow up. J Neuroimmunol. 2021 Jul;356:577590.

35. Gagarkin DA, Dombrowski KE, Thakar KB, DePetrillo JC. Acute inflammatory demyelinating polyneuropathy or Guillain-Barré syndrome associated with COVID-19: a case report. J Med Case Rep. 2021 Dec 28;15(1):219.

36. Pujol Lucas C, Lobaco Soler M, Roca Rabionet C, Alfonso Martín S, Aragonés Pascual JM, Espaulella i Panicot J. Síndrome de Guillain-Barré como probable primer síntoma de síndrome respiratorio agudo severo Coronavirus 2 en una paciente anciana. Rev Esp Geriatr Gerontol. 2021 Jul;56(4):252–3.

37. Coll C, Tessier M, Vandendries C, Seror P. Neuralgic amyotrophy and COVID-19 infection: 2 cases of spinal accessory nerve palsy. Joint Bone Spine. 2021 Oct;88(5):105196.

38. Yiu AC, Hussain A, Okonkwo UA, Villacorta-Lyew R, McMahon MJ, Blattner M. Guillain–Barre Syndrome Associated With COVID-19 Pneumonia—The First Documented Case in a U.S. Military Intensive Care Unit. Mil Med. 2021 Apr 21;

39. Anilkumar A, Tan E, Cleaver J, Morrison HD. Isolated abducens nerve palsy in a patient with asymptomatic SARS-CoV-2 infection. Journal of Clinical Neuroscience. 2021 Jul;89:65–7.

40. Ates MF, Karsidag S. Ptosis and COVID-19. Prim Care Companion CNS Disord. 2021 Apr 8;23(2).

41. Kerstens J, Deschuytere L, Schotsmans K, Maréchal E. Bilateral peripheral facial palsy following asymptomatic COVID-19 infection: a case report. Acta Neurol Belg. 2021 Jun 5;121(3):815–6.

42. Douedi S, Naser H, Mazahir U, Hamad AI, Sedarous M. Third Cranial Nerve Palsy Due to COVID-19 Infection. Cureus. 2021 Apr 3;

43. Dias FA, Cunha ALN, Pantoja PMP, Moreira CL, Tomaselli PJ, Zanon Zotin MC, et al. Acute Inflammatory Painful Polyradiculoneuritis. Neurol Clin Pract. 2021 Apr;11(2):e205–7.

44. Ebrahimzadeh SA, Ghoreishi A, Rahimian N. Guillain-Barré Syndrome Associated With COVID-19. Neurol Clin Pract. 2021 Apr;11(2):e196–8.

45. Sharma A, Kudchadkar U, Shirodkar R, Usgaonkar US, Naik A. Unilateral inferior altitudinal visual field defect related to COVID-19. Indian J Ophthalmol. 2021;69(4):989.

46. Brown M, Petrassi A, Bureau BL, Khan N, Jha P. SARS-CoV-2-Associated Guillain-Barre Syndrome Obscured by Diabetes Mellitus Peripheral Neuropathy. Cureus. 2021 Mar 31;

47. Mokhashi N, Narla G, Marchionni C. Guillain-Barre Syndrome in a Patient With Asymptomatic Coronavirus Disease 2019 Infection and Major Depressive Disorder. Cureus. 2021 Mar 28;

48. Yakoby J, Litvak I, Yu E. Guillain-Barré Syndrome after Novel Coronavirus Disease 2019. J Emerg Med. 2021 Oct;61(4):e67–70.

49. Han CY, Tarr AM, Gewirtz AN, Kaunzner UW, Roy-Burman P, Cutler TS, et al. Brachial plexopathy as a complication of COVID-19. BMJ Case Rep. 2021 Mar 25;14(3):e237459.

50. Mackenzie N, Lopez-Coronel E, Dau A, Maloof D, Mattar S, Garcia JT, et al. Concomitant Guillain-Barre syndrome with COVID-19: a case report. BMC Neurol. 2021 Dec 23;21(1):135.

51. Rodríguez-Rodríguez MS, Romero-Castro RM, Alvarado-de la Barrera C, González-Cannata MG, García-Morales AK, Ávila-Ríos S. Optic neuritis following SARS-CoV-2 infection. J Neurovirol. 2021 Apr 23;27(2):359–63.

52. Ismail II, Abdelnabi EA, Al-Hashel JY, Alroughani R, Ahmed SF. Neuralgic amyotrophy associated with COVID-19 infection: a case report and review of the literature. Neurological Sciences. 2021 Jun 20;42(6):2161–5.

53. Svačina MKR, Kohle F, Sprenger A, Lehmann HC. Could symptom overlap of COVID-19 and Guillain–Barré syndrome mask an epidemiological association? J Neurol. 2021 Oct 17;268(10):3595–7.

54. Amanat M, Rezaei N, Roozbeh M, Shojaei M, Tafakhori A, Zoghi A, et al. Neurological manifestations as the predictors of severity and mortality in hospitalized individuals with COVID-19: a multicenter prospective clinical study. BMC Neurol. 2021 Dec 16;21(1):116.

55. Oke IO, Oladunjoye OO, Oladunjoye AO, Paudel A, Zimmerman R. Bell’s Palsy as a Late Neurologic Manifestation of COVID-19 Infection. Cureus. 2021 Mar 14;

56. de Francisco Moure J, Torres Ramón I, Almárcegui Lafita C. Acute Polyneuropathy in an Outpatient Context During the SARS-CoV-2 Pandemic: A Brief Case Serie Report. SN Compr Clin Med. 2021 May 12;3(5):1182–4.

57. Beckers E, Chouvel P, Cassetto V, Mustin V. Sudden sensorineural hearing loss in COVID‐19: A case report and literature review. Clin Case Rep. 2021 Apr 11;9(4):2300–4.

58. Singh R, Shiza ST, Saadat R, Dawe M, Rehman U. Association of Guillain-Barre Syndrome With COVID-19: A Case Report and Literature Review. Cureus. 2021 Mar 11;

59. Molina‐Gil J, González‐Fernández L, García‐Cabo C. Trigeminal neuralgia as the sole neurological manifestation of COVID‐19: A case report. Headache: The Journal of Head and Face Pain. 2021 Mar 22;61(3):560–2.

60. Kumar V, Narayanan P, Shetty S, Mohammed AP. Lower motor neuron facial palsy in a postnatal mother with COVID-19. BMJ Case Rep. 2021 Mar 1;14(3):e240267.

61. Todisco M, Alfonsi E, Arceri S, Bertino G, Robotti C, Albergati M, et al. Isolated bulbar palsy after SARS-CoV-2 infection. Lancet Neurol. 2021 Mar;20(3):169–70.

62. Kaya Tutar N, Kale N, Tugcu B. Adie-Holmes syndrome associated with COVID-19 infection: A case report. Indian J Ophthalmol. 2021;69(3):773.

63. Bartoli A, Gitto S, Sighinolfi P, Cursaro C, Andreone P. Primary biliary cholangitis associated with SARS-CoV-2 infection. J Hepatol. 2021 May;74(5):1245–6.

64. Neo WL, Ng JCF, Iyer NG. The great pretender—Bell’s palsy secondary to SARS‐CoV‐2? Clin Case Rep. 2021 Mar 12;9(3):1175–7.

65. Türk Börü Ü, Köseoğlu Toksoy C, Bölük C, Demirbaş H, Yılmaz AÇ. A case of Guillain-Barré syndrome related to COVID-19 infection. International Journal of Neuroscience. 2021 Feb 12;1–3.

66. Meshref M, A. Alshammari H, Khairat SM, Khan R, Khan I. Guillain-Barre Syndrome Associated With COVID-19 Infection: A Case Report With Review of Literature. Cureus. 2021 Feb 3;

67. Tekin AB, Zanapalioglu U, Gulmez S, Akarsu I, Yassa M, Tug N. Guillain Barre Syndrome following delivery in a pregnant woman infected with SARS-CoV-2. Journal of Clinical Neuroscience. 2021 Apr;86:190–2.

68. Rodrigo-Armenteros P, Uterga-Valiente JM, Zabala-Del-Arco J, Taramundi-Argüeso S, Antón-Méndez L, Gómez-Muga JJ, et al. Optic neuropathy in a patient with COVID-19 infection. Acta Neurol Belg. 2022 Jun 2;122(3):789–91.

69. Acute Flaccid Quadriparesis in a Recovering COVID-19 Patient: A Clinical Dilemma. Indian Journal of Critical Care Medicine. 2021 Feb 22;25(2):238–9.

70. Goel K, Diwan S, Kohli S, Sachdeva HC, Mustafi SM. Neurological Manifestations of COVID-19: A Series of Seven Cases. Indian Journal of Critical Care Medicine. 2021 Feb 22;25(2):219–23.

71. François J, Collery AS, Hayek G, Sot M, Zaidi M, Lhuillier L, et al. Coronavirus Disease 2019–Associated Ocular Neuropathy With Panuveitis. JAMA Ophthalmol. 2021 Feb 1;139(2):247.

72. Bahouth S, Chuang K, Olson L, Rosenthal D. COVID-19 related muscle denervation atrophy. Skeletal Radiol. 2021 Aug 31;50(8):1717–21.

73. de Gennaro R, Gastaldo E, Tamborino C, Baraldo M, Casula N, Pedrali M, et al. Selective cranial multineuritis in severe COVID-19 pneumonia: two cases and literature review. Neurological Sciences. 2021 May 30;42(5):1643–8.

74. Raahimi MM, Kane A, Moore CE, Alareed AW. Late onset of Guillain-Barré syndrome following SARS-CoV-2 infection: part of ‘long COVID-19 syndrome’? BMJ Case Rep. 2021 Jan 18;14(1):e240178.

75. Zubair AS, Zubair AS, Desai K, Abulaban A, Roy B. Guillain-Barré Syndrome as a Complication of COVID-19. Cureus. 2021 Jan 14;

76. Dufour C, Co TK, Liu A. GM1 ganglioside antibody and COVID-19 related Guillain Barre Syndrome – A case report, systemic review and implication for vaccine development. Brain Behav Immun Health. 2021 Mar;12:100203.

77. Dahl EH, Mosevoll KA, Cramariuc D, Vedeler CA, Blomberg B. COVID-19 myocarditis and postinfection Bell’s palsy. BMJ Case Rep. 2021 Jan 11;14(1):e240095.

78. Francis JE. Abducens Palsy and Anosmia Associated with COVID-19: A Case Report. British and Irish Orthoptic Journal. 2021 Jan 18;17(1):8–12.

79. Khedr EM, Shoyb A, Mohamed KO, Karim AA, Saber M. Case Report: Guillain–Barré Syndrome Associated With COVID-19. Front Neurol. 2021 Jun 22;12.

80. Singhai A, Budhiraja A. Guillain–Barre Syndrome following SARS-COVID-19 Infection: A Case Report from India. Case Rep Infect Dis. 2021 May 27;2021:1–4.

81. Žorić L, Rajović-Mrkić I, Čolak E, Mirić D, Kisić B. Optic Neuritis in a Patient with Seropositive Myelin Oligodendrocyte Glycoprotein Antibody During the Post-COVID-19 Period. Int Med Case Rep J. 2021 May;Volume 14:349–55.

82. Halalau A, Halalau M, Carpenter C, Abbas AE, Sims M. Vestibular neuritis caused by severe acute respiratory syndrome coronavirus 2 infection diagnosed by serology: Case report. SAGE Open Med Case Rep. 2021 Jan 13;9:2050313X2110132.

83. Julie Paradis, Hendrik Kevers, Soufiane Chebli, Serge Blum, Pascal Reper. Unusual Presentation of COVID-19 Disease Leading to Respiratory Distress and Coma. Eur J Case Rep Intern Med. 2021 Apr 7;(Vol 8 No 4).

84. Ansari B, Hemasian H. Peculiar Presentation of COVID-19: A Case Report of Concurrent Stroke and Guillain–Barré Syndrome. Case Rep Neurol Med. 2021 Feb 15;2021:1–4.

85. Bastola A, Sah R, Nepal G, Gajurel BP, Rajbhandari SK, Chalise BS, et al. Bell’s palsy as a possible neurological complication of COVID‐19: A case report. Clin Case Rep. 2021 Feb 8;9(2):747–50.

86. Manganotti P, Bellavita G, D’Acunto L, Tommasini V, Fabris M, Sartori A, et al. Clinical neurophysiology and cerebrospinal liquor analysis to detect Guillain‐Barré syndrome and polyneuritis cranialis in COVID‐19 patients: A case series. J Med Virol. 2021 Feb 27;93(2):766–74.

87. Agosti E, Giorgianni A, D’Amore F, Vinacci G, Balbi S, Locatelli D. Is Guillain-Barrè syndrome triggered by SARS-CoV-2? Case report and literature review. Neurological Sciences. 2021 Feb 9;42(2):607–12.

88. Li CW, Syue LS, Tsai YS, Li MC, Lo CL, Tsai CS, et al. Anosmia and olfactory tract neuropathy in a case of COVID-19. Journal of Microbiology, Immunology and Infection. 2021 Feb;54(1):93–6.

89. Aasfara J, Hajjij A, Bensouda H, Ouhabi H, Benariba F. A unique association of bifacial weakness, paresthesia and vestibulocochlear neuritis as post COVID 19 manifestation in pregnant women. Pan African Medical Journal. 2021;38.

90. Colonna S, Sciumé L, Giarda F, Innocenti A, Beretta G, Dalla Costa D. Case Report: Postacute Rehabilitation of Guillain-Barré Syndrome and Cerebral Vasculitis-Like Pattern Accompanied by SARS-CoV-2 Infection. Front Neurol. 2021 Jan 7;11.

91. Eslamian F, Taleschian-Tabrizi N, Izadseresht B, Shakouri SK, Gholian S, Rahbar M. Electrophysiologic findings in patients with COVID-19 and quadriparesia in the northwest of Iran, A case series study and literature review. Caspian J Intern Med. 2021;12(Suppl 2):S451–9.

92. Kumar H, Chakraborty N. Intravenous Immunoglobulin may Reverse Multisystem Inflammation in COVID-19 Pneumonitis and Guillain&amp;ndash;Barr&amp;eacute; Syndrome. Indian Journal of Critical Care Medicine. 2020 Dec 26;24(12):1264–8.

93. Moyano A JR, Mejía Torres S, Espinosa J. Vagus nerve neuropathy related to SARS COV-2 infection. IDCases. 2021;26:e01242.

94. Sisniega DC, Reynolds AS. Severe Neurologic Complications of SARS-CoV-2. Curr Treat Options Neurol. 2021 May 31;23(5):14.

95. Bueso T, Montalvan V, Lee J, Gomez J, Ball S, Shoustari A, et al. Guillain-Barre Syndrome and COVID-19: A case report. Clin Neurol Neurosurg. 2021 Jan;200:106413.

96. Abolmaali M, Heidari M, Zeinali M, Moghaddam P, Ramezani Ghamsari M, Jamshidi Makiani M, et al. Guillain–Barré syndrome as a parainfectious manifestation of SARS-CoV-2 infection: A case series. Journal of Clinical Neuroscience. 2021 Jan;83:119–22.

97. Karimi-Galougahi M, Yousefi-Koma A, Raygani N, Bakhshayeshkaram M, Haseli S. 18FDG-PET/CT Assessment of COVID-19-Induced Bell’s Palsy. Acad Radiol. 2021 Jan;28(1):144–5.

98. Nanda S, Handa R, Prasad A, Anand R, Zutshi D, Dass SK, et al. Covid-19 associated Guillain-Barre Syndrome: Contrasting tale of four patients from a tertiary care centre in India. Am J Emerg Med. 2021 Jan;39:125–8.

99. Gogia B, Gil Guevara A, Rai PK, Fang X. A case of COVID-19 with multiple cranial neuropathies. International Journal of Neuroscience. 2020 Dec 30;1–3.

100. Soliman SB, Klochko CL, Dhillon MK, Vandermissen NR, van Holsbeeck MT. Peripheral Polyneuropathy Associated with COVID-19 in Two Patients: A Musculoskeletal Ultrasound Case Report. J Med Ultrasound. 28(4):249–52.

101. Judge C, Moheb N, Castro Apolo R, Dupont JL, Gessner ML, Yacoub HA. Facial Diplegia as a Rare Late Neurologic Manifestation of SARS-CoV-2 Infection. J Neurol Res. 2020;10(6):235–6.

102. Petrelli C, Scendoni R, Paglioriti M, Logullo FO. Acute Motor Axonal Neuropathy Related to COVID-19 Infection: A New Diagnostic Overview. J Clin Neuromuscul Dis. 2020 Dec;22(2):120–1.

103. Raharimanantsoa OL, Razakarivony FA, Andriamiadanalisoa AO, Rajaona RA, Rakotoarisoa R, Randrianarimanana SER, et al. Multinévrite oculaire dans le cadre d’une infection COVID-19. J Fr Ophtalmol. 2020 Dec;43(10):e351–3.

104. Ghosh R, Roy D, Sengupta S, Benito-León J. Autonomic dysfunction heralding acute motor axonal neuropathy in COVID-19. J Neurovirol. 2020 Dec 11;26(6):964–6.

105. Cabañes-Martínez L, Villadóniga M, González-Rodríguez L, Araque L, Díaz-Cid A, Ruz-Caracuel I, et al. Neuromuscular involvement in COVID-19 critically ill patients. Clinical Neurophysiology. 2020 Dec;131(12):2809–16.

106. Lima MA, Silva MTT, Soares CN, Coutinho R, Oliveira HS, Afonso L, et al. Peripheral facial nerve palsy associated with COVID-19. J Neurovirol. 2020 Dec 2;26(6):941–4.

107. Costa Martins D, Branco Ribeiro S, Jesus Pereira I, Mestre S, Rios J. Unilateral Hypoglossal Nerve Palsy as a COVID-19 Sequel. Am J Phys Med Rehabil. 2020 Dec;99(12):1096–8.

108. Greer CE, Bhatt JM, Oliveira CA, Dinkin MJ. Isolated Cranial Nerve 6 Palsy in 6 Patients With COVID-19 Infection. Journal of Neuro-Ophthalmology. 2020 Dec;40(4):520–2.

109. Ordás CM, Villacieros-Álvarez J, Pastor-Vivas AI, Corrales-Benítez Á. Concurrent tonic pupil and trochlear nerve palsy in COVID-19. J Neurovirol. 2020 Dec 10;26(6):970–2.

110. Civardi C, Collini A, Geda DJ, Geda C. Antiganglioside antibodies in Guillain-Barré syndrome associated with SARS-CoV-2 infection. J Neurol Neurosurg Psychiatry. 2020 Dec;91(12):1361–2.

111. Derollez C, Alberto T, Leroi I, Mackowiak M ‐A., Chen Y. Facial nerve palsy: an atypical clinical manifestation of COVID‐19 infection in a family cluster. Eur J Neurol. 2020 Dec 8;27(12):2670–2.

112. Virgo J, Mohamed M. Paracentral acute middle maculopathy and acute macular neuroretinopathy following SARS-CoV-2 infection. Eye. 2020 Dec 3;34(12):2352–3.

113. Karimi-Galougahi M, Naeini AS, Raad N, Mikaniki N, Ghorbani J. Vertigo and hearing loss during the COVID-19 pandemic – is there an association? Acta Otorhinolaryngologica Italica. 2020 Dec;40(6):463–5.

114. Gale A, Sabaretnam S, Lewinsohn A. Guillain-Barré syndrome and COVID-19: association or coincidence. BMJ Case Rep. 2020 Nov 30;13(11):e239241.

115. Vanaparthy R, Malayala S v, Balla M. COVID-19-Induced Vestibular Neuritis, Hemi-Facial Spasms and Raynaud’s Phenomenon: A Case Report. Cureus. 2020 Nov 28;

116. Kushwaha S, Seth V, Bapat P, R K, Chaturvedi M, Gupta R, et al. Neurological Associations of COVID-19—Do We Know Enough: A Tertiary Care Hospital Based Study. Front Neurol. 2020 Nov 24;11.

117. Liberatore G, de Santis T, Doneddu PE, Gentile F, Albanese A, Nobile-Orazio E. Clinical Reasoning: A case of COVID-19–associated pharyngeal-cervical-brachial variant of Guillain-Barré syndrome. Neurology. 2020 Nov 24;95(21):978–83.

118. McDonnell EP, Altomare NJ, Parekh YH, Gowda RC, Parikh PD, Lazar MH, et al. COVID-19 as a Trigger of Recurrent Guillain–Barré Syndrome. Pathogens. 2020 Nov 19;9(11):965.

119. Kanjwal K, Jamal S, Kichloo A, Grubb B. New-onset Postural Orthostatic Tachycardia Syndrome Following Coronavirus Disease 2019 Infection. Journal of Innovations in Cardiac Rhythm Management. 2020 Nov 1;11(11):4302–4.

120. Bureau BL, Obeidat A, Dhariwal MS, Jha P. Peripheral Neuropathy as a Complication of SARS-Cov-2. Cureus. 2020 Nov 12;

121. Othenin-Girard A, Regamey J, Lamoth F, Horisberger A, Glampedakis E, Epiney JB, et al. Multisystem inflammatory syndrome with refractory cardiogenic shock due to acute myocarditis and mononeuritis multiplex after SARS-CoV-2 infection in an adult. Swiss Med Wkly. 2020 Nov 11;

122. Lowery MM, Taimur Malik M, Seemiller J, Tsai CS. Atypical Variant of Guillain Barre Syndrome in a Patient with COVID-19. The Journal of Critical Care Medicine. 2020 Nov 7;6(4):231–6.

123. Boostani R, Talab FR, Meibodi NT, Zemorshidi F. COVID-19 associated with sensorimotor polyradiculoneuropathy and skin lesions: A case report. J Neuroimmunol. 2021 Jan;350:577434.

124. Groiss SJ, Balloff C, Elben S, Brandenburger T, Müttel T, Kindgen-Milles D, et al. Prolonged Neuropsychological Deficits, Central Nervous System Involvement, and Brain Stem Affection After COVID-19—A Case Series. Front Neurol. 2020 Nov 5;11.

125. Senel M, Abu‐Rumeileh S, Michel D, Garibashvili T, Althaus K, Kassubek J, et al. Miller‐Fisher syndrome after COVID‐19: neurochemical markers as an early sign of nervous system involvement. Eur J Neurol. 2020 Nov 13;27(11):2378–80.

126. Bracaglia M, Naldi I, Govoni A, Brillanti Ventura D, de Massis P. Acute inflammatory demyelinating polyneuritis in association with an asymptomatic infection by SARS-CoV-2. J Neurol. 2020 Nov 25;267(11):3166–8.

127. Faucher A, Rey PA, Aguadisch E, Degos B. Isolated post SARS-CoV-2 diplopia. J Neurol. 2020 Nov 12;267(11):3128–9.

128. Esteban Molina A, Mata Martínez M, Sánchez Chueca P, Carrillo López A, Sancho Val I, Sanjuan-Villarreal TA. Síndrome de Guillain-Barré asociado a infección por COVID-19. Med Intensiva. 2020 Nov;44(8):513–4.

129. Chan JL, Ebadi H, Sarna JR. Guillain-Barré Syndrome with Facial Diplegia Related to SARS-CoV-2 Infection. Canadian Journal of Neurological Sciences / Journal Canadien des Sciences Neurologiques. 2020 Nov 29;47(6):852–4.

130. Khaja M, Gomez GPR, Santana Y, Hernandez N, Haider A, Lara JLP, et al. A 44-Year-Old Hispanic Man with Loss of Taste and Bilateral Facial Weakness Diagnosed with Guillain-Barré Syndrome and Bell’s Palsy Associated with SARS-CoV-2 Infection Treated with Intravenous Immunoglobulin. American Journal of Case Reports. 2020 Sep 23;21.

131. Hirayama T, Hongo Y, Kaida K, Kano O. Guillain-Barré syndrome after COVID-19 in Japan. BMJ Case Rep. 2020 Oct 29;13(10):e239218.

132. García-Manzanedo S, López de la Oliva Calvo L, Ruiz Álvarez L. Guillain-Barré syndrome after Covid-19 infection. Medicina Clínica (English Edition). 2020 Oct;155(8):366.

133. Belghmaidi S, Nassih H, Boutgayout S, el Fakiri K, el Qadiry R, Hajji I, et al. Third Cranial Nerve Palsy Presenting with Unilateral Diplopia and Strabismus in a 24-Year-Old Woman with COVID-19. American Journal of Case Reports. 2020 Sep 7;21.

134. Faqihi F, Alharthy A, Memish ZA, Kutsogiannis DJ, Brindley PG, Karakitsos D. Peripheral neuropathy in severe COVID‐19 resolved with therapeutic plasma exchange. Clin Case Rep. 2020 Dec 7;8(12):3233–8.

135. RIBEIRO BN de F, MARCHIORI E. Facial palsy as a neurological complication of SARS-CoV-2. Arq Neuropsiquiatr. 2020 Oct;78(10):667–667.

136. Agha Abbaslou M, Karbasi M, Mozhdehipanah H. A Rare Axonal Variant of Guillain-Barré Syndrome as a Neurological Complication of COVID-19 Infection. Arch Iran Med. 2020 Oct 1;23(10):718–21.

137. Lang B, Hintze J, Conlon B. Coronavirus disease 2019 and sudden sensorineural hearing loss. J Laryngol Otol. 2020 Nov 1;134(11):1026–8.

138. Redondo Urda MJ, Rodríguez Peguero FJ, Pérez Gil O, del Valle Sánchez M, Carrera Izquierdo M. SARS-CoV-2, nuevo agente causal del síndrome de Guillain-Barré. Rev Neurol. 2020;71(07):275.

139. Diez-Porras L, Vergés E, Gil F, Vidal MJ, Massons J, Arboix A. Guillain-Barré-Strohl syndrome and COVID-19: Case report and literature review. Neuromuscular Disorders. 2020 Oct;30(10):859–61.

140. Abrams RMC, Kim BD, Markantone DM, Reilly K, Paniz-Mondolfi AE, Gitman MR, et al. Severe rapidly progressive Guillain-Barré syndrome in the setting of acute COVID-19 disease. J Neurovirol. 2020 Oct 27;26(5):797–9.

141. Siepmann T, Kitzler HH, Lueck C, Platzek I, Reichmann H, Barlinn K. Neuralgic amyotrophy following infection with <scp>SARS‐CoV</scp> ‐2. Muscle Nerve. 2020 Oct 10;62(4).

142. Korem S, Gandhi H, Dayag DB. Guillain-Barré syndrome associated with COVID-19 disease. BMJ Case Rep. 2020 Sep 21;13(9):e237215.

143. Ameer N, Shekhda KM, Cheesman A. Guillain-Barré syndrome presenting with COVID-19 infection. BMJ Case Rep. 2020 Sep 14;13(9):e236978.

144. Granger A, Omari M, Jakubowska-Sadowska K, Boffa M, Zakin E. SARS-CoV-2–Associated Guillain–Barre Syndrome With Good Response to Plasmapheresis. J Clin Neuromuscul Dis. 2020 Sep;22(1):58–9.

145. Wada S, Nagasaki Y, Arimizu Y, Shimo M, Matsukuma Y, Okamoto M, et al. Neurological Disorders Identified during Treatment of a SARS-CoV-2 Infection. Internal Medicine. 2020 Sep 1;59(17):2187–9.

146. Pascual-Goñi E, Fortea J, Martínez-Domeño A, Rabella N, Tecame M, Gómez-Oliva C, et al. COVID-19-associated ophthalmoparesis and hypothalamic involvement. Neurology - Neuroimmunology Neuroinflammation. 2020 Sep 25;7(5):e823.

147. Hutchins KL, Jansen JH, Comer AD, Scheer RV, Zahn GS, Capps AE, et al. COVID-19–Associated Bifacial Weakness with Paresthesia Subtype of Guillain-Barré Syndrome. American Journal of Neuroradiology. 2020 Jun 25;

148. Kilinc D, Pasch S, Doets AY, Jacobs BC, Vliet J, Garssen MPJ. Guillain–Barré syndrome after SARS‐CoV‐2 infection. Eur J Neurol. 2020 Sep 20;27(9):1757–8.

149. Oguz‐Akarsu E, Ozpar R, Mirzayev H, Acet‐Ozturk NA, Hakyemez B, Ediger D, et al. <scp>Guillain‐Barré</scp> Syndrome in a Patient With Minimal Symptoms of <scp>COVID</scp> ‐19 Infection. Muscle Nerve. 2020 Sep 4;62(3).

150. Lascano AM, Epiney J ‐B., Coen M, Serratrice J, Bernard‐Valnet R, Lalive PH, et al. SARS‐CoV‐2 and Guillain–Barré syndrome: AIDP variant with a favourable outcome. Eur J Neurol. 2020 Sep 20;27(9):1751–3.

151. Bigaut K, Mallaret M, Baloglu S, Nemoz B, Morand P, Baicry F, et al. Guillain-Barré syndrome related to SARS-CoV-2 infection. Neurology - Neuroimmunology Neuroinflammation. 2020 Sep 27;7(5):e785.

152. Fernández-Domínguez J, Ameijide-Sanluis E, García-Cabo C, García-Rodríguez R, Mateos V. Miller–Fisher-like syndrome related to SARS-CoV-2 infection (COVID 19). J Neurol. 2020 Sep 26;267(9):2495–6.

153. Atakla HG, Noudohounsi MMUD, Sacca H, Tassiou NRA, Noudohounsi WC, Houinato DS. Acute Guillain Barré polyradiculoneuritis indicative of COVID-19 infection: a case report. Pan African Medical Journal. 2020;35.

154. Goh Y, Beh DLL, Makmur A, Somani J, Chan ACY. Pearls &amp; Oy-sters: Facial nerve palsy in COVID-19 infection. Neurology. 2020 Aug 25;95(8):364–7.

155. Ray A. Miller Fisher syndrome and COVID-19: is there a link? BMJ Case Rep. 2020 Aug 11;13(8):e236419.

156. Figueiredo R, Falcão V, Pinto MJ, Ramalho C. Peripheral facial paralysis as presenting symptom of COVID-19 in a pregnant woman. BMJ Case Rep. 2020 Aug 11;13(8):e237146.

157. Dinkin M, Gao V, Kahan J, Bobker S, Simonetto M, Wechsler P, et al. COVID-19 presenting with ophthalmoparesis from cranial nerve palsy. Neurology. 2020 Aug 4;95(5):221–3.

158. Gutiérrez-Ortiz C, Méndez-Guerrero A, Rodrigo-Rey S, San Pedro-Murillo E, Bermejo-Guerrero L, Gordo-Mañas R, et al. Miller Fisher syndrome and polyneuritis cranialis in COVID-19. Neurology. 2020 Aug 4;95(5):e601–5.

159. Naddaf E, Laughlin RS, Klein CJ, Toledano M, Theel ES, Binnicker MJ, et al. Guillain-Barré Syndrome in a Patient With Evidence of Recent SARS-CoV-2 Infection. Mayo Clin Proc. 2020 Aug;95(8):1799–801.

160. Falcone MM, Rong AJ, Salazar H, Redick DW, Falcone S, Cavuoto KM. Acute abducens nerve palsy in a patient with the novel coronavirus disease (COVID-19). Journal of American Association for Pediatric Ophthalmology and Strabismus. 2020 Aug;24(4):216–7.

161. Kilic O, Kalcioglu MT, Cag Y, Tuysuz O, Pektas E, Caskurlu H, et al. Could sudden sensorineural hearing loss be the sole manifestation of COVID-19? An investigation into SARS-COV-2 in the etiology of sudden sensorineural hearing loss. International Journal of Infectious Diseases. 2020 Aug;97:208–11.

162. Aoyagi Y, Ohashi M, Funahashi R, Otaka Y, Saitoh E. Oropharyngeal Dysphagia and Aspiration Pneumonia Following Coronavirus Disease 2019: A Case Report. Dysphagia. 2020 Aug 12;35(4):545–8.

163. Manganotti P, Pesavento V, Buoite Stella A, Bonzi L, Campagnolo E, Bellavita G, et al. Miller Fisher syndrome diagnosis and treatment in a patient with SARS-CoV-2. J Neurovirol. 2020 Aug 11;26(4):605–6.

164. Su XW, Palka S v., Rao RR, Chen FS, Brackney CR, Cambi F. <scp>SARS‐CoV</scp> ‐2–associated <scp>Guillain‐Barré</scp> syndrome with dysautonomia. Muscle Nerve. 2020 Aug;62(2).

165. Tiet MY, AlShaikh N. Guillain-Barré syndrome associated with COVID-19 infection: a case from the UK. BMJ Case Rep. 2020 Jul 8;13(7):e236536.

166. Paybast S, Gorji R, Mavandadi S. Guillain-Barré Syndrome as a Neurological Complication of Novel COVID-19 Infection. Neurologist. 2020 Jul;25(4):101–3.

167. Casas Peña E, Barbosa del Olmo A, Rubio García E, Cebrián J, Díaz Pérez C, de la Fuente E, et al. Parálisis facial periférica aislada en un paciente con COVID-19. Rev Neurol. 2020;71(01):40.

168. Guijarro-Castro C, Rosón-González M, Abreu A, García-Arratibel A, Ochoa-Mulas M. Síndrome de Guillain-Barré tras infección por SARS-CoV-2. Comentarios tras la publicación de 16 nuevos casos. Neurología. 2020 Jul;35(6):412–5.

169. Sancho-Saldaña A, Lambea-Gil Á, Liesa JLC, Caballo MRB, Garay MH, Celada DR, et al. Guillain–Barré syndrome associated with leptomeningeal enhancement following SARS-CoV-2 infection. Clinical Medicine. 2020 Jul;20(4):e93–4.

170. Assini A, Benedetti L, di Maio S, Schirinzi E, del Sette M. New clinical manifestation of COVID-19 related Guillain-Barrè syndrome highly responsive to intravenous immunoglobulins: two Italian cases. Neurological Sciences. 2020 Jul 28;41(7):1657–8.

171. Lantos JE, Strauss SB, Lin E. COVID-19–Associated Miller Fisher Syndrome: MRI Findings. American Journal of Neuroradiology. 2020 Jul;41(7):1184–6.

172. Arnaud S, Budowski C, Ng Wing Tin S, Degos B. Post SARS-CoV-2 Guillain-Barré syndrome. Clinical Neurophysiology. 2020 Jul;131(7):1652–4.

173. Juliao Caamaño DS, Alonso Beato R. Facial diplegia, a possible atypical variant of Guillain-Barré Syndrome as a rare neurological complication of SARS-CoV-2. Journal of Clinical Neuroscience. 2020 Jul;77:230–2.

174. Pfefferkorn T, Dabitz R, von Wernitz-Keibel T, Aufenanger J, Nowak-Machen M, Janssen H. Acute polyradiculoneuritis with locked-in syndrome in a patient with Covid-19. J Neurol. 2020 Jul 12;267(7):1883–4.

175. Coen M, Jeanson G, Culebras Almeida LA, Hübers A, Stierlin F, Najjar I, et al. Guillain-Barré syndrome as a complication of SARS-CoV-2 infection. Brain Behav Immun. 2020 Jul;87:111–2.

176. Alberti P, Beretta S, Piatti M, Karantzoulis A, Piatti ML, Santoro P, et al. Guillain-Barré syndrome related to COVID-19 infection. Neurology - Neuroimmunology Neuroinflammation. 2020 Jul 29;7(4):e741.

177. Padroni M, Mastrangelo V, Asioli GM, Pavolucci L, Abu-Rumeileh S, Piscaglia MG, et al. Guillain-Barré syndrome following COVID-19: new infection, old complication? J Neurol. 2020 Jul 24;267(7):1877–9.

178. Malayala S v, Raza A. A Case of COVID-19-Induced Vestibular Neuritis. Cureus. 2020 Jun 30;

179. Toscano G, Palmerini F, Ravaglia S, Ruiz L, Invernizzi P, Cuzzoni MG, et al. Guillain–Barré Syndrome Associated with SARS-CoV-2. New England Journal of Medicine. 2020 Jun 25;382(26):2574–6.

180. Farzi MA, Ayromlou H, Jahanbakhsh N, Bavil PH, Janzadeh A, Shayan FK. Guillain-Barré syndrome in a patient infected with SARS-CoV-2, a case report. J Neuroimmunol. 2020 Sep;346:577294.

181. Webb S, Wallace VC, Martin-Lopez D, Yogarajah M. Guillain-Barré syndrome following COVID-19: a newly emerging post-infectious complication. BMJ Case Rep. 2020 Jun 14;13(6):e236182.

182. Rana S, Lima AA, Chandra R, Valeriano J, Desai T, Freiberg W, et al. Novel Coronavirus (COVID-19)-Associated Guillain–Barré Syndrome: Case Report. J Clin Neuromuscul Dis. 2020 Jun;21(4):240–2.

183. Ottaviani D, Boso F, Tranquillini E, Gapeni I, Pedrotti G, Cozzio S, et al. Early Guillain-Barré syndrome in coronavirus disease 2019 (COVID-19): a case report from an Italian COVID-hospital. Neurological Sciences. 2020 Jun 12;41(6):1351–4.

184. Scheidl E, Canseco DD, Hadji‐Naumov A, Bereznai B. <scp>Guillain‐Barr</scp> é syndrome during <scp>SARS‐CoV</scp> ‐2 pandemic: A case report and review of recent literature. Journal of the Peripheral Nervous System. 2020 Jun 26;25(2):204–7.

185. el Otmani H, el Moutawakil B, Rafai MA, el Benna N, el Kettani C, Soussi M, et al. Covid-19 and Guillain-Barré syndrome: More than a coincidence! Rev Neurol (Paris). 2020 Jun;176(6):518–9.

186. Abdelnour L, Eltahir Abdalla M, Babiker S. COVID 19 infection presenting as motor peripheral neuropathy. Journal of the Formosan Medical Association. 2020 Jun;119(6):1119–20.

187. Camdessanche JP, Morel J, Pozzetto B, Paul S, Tholance Y, Botelho-Nevers E. COVID-19 may induce Guillain–Barré syndrome. Rev Neurol (Paris). 2020 Jun;176(6):516–8.

188. Sedaghat Z, Karimi N. Guillain Barre syndrome associated with COVID-19 infection: A case report. Journal of Clinical Neuroscience. 2020 Jun;76:233–5.

189. Francois Maurier, Benoit Godbert, Julie Perrin. Respiratory Distress in SARS-CoV-2 without Lung Damage: Phrenic Paralysis Should Be Considered in COVID-19 Infection. Eur J Case Rep Intern Med. 2020 May 21;(Vol 7 No 6).

190. Ghiasvand F, Ghadimi M, Ghadimi F, Safarpour S, Hosseinzadeh R, SeyedAlinaghi S. Symmetrical polyneuropathy in coronavirus disease 2019 (COVID-19). IDCases. 2020;21:e00815.

191. Marta-Enguita J, Rubio-Baines I, Gastón-Zubimendi I. Síndrome de Guillain-Barré fatal tras infección por el virus SARS-CoV-2. Neurología. 2020 May;35(4):265–7.

192. Velayos Galán A, del Saz Saucedo P, Peinado Postigo F, Botia Paniagua E. Síndrome de Guillain-Barré asociado a infección por SARS-CoV-2. Neurología. 2020 May;35(4):268–9.

193. Zhao H, Shen D, Zhou H, Liu J, Chen S. Guillain-Barré syndrome associated with SARS-CoV-2 infection: causality or coincidence? Lancet Neurol. 2020 May;19(5):383–4.

194. Wei H, Yin H, Huang M, Guo Z. The 2019 novel cornoavirus pneumonia with onset of oculomotor nerve palsy: a case study. J Neurol. 2020 May 25;267(5):1550–3.

195. Virani A, Rabold E, Hanson T, Haag A, Elrufay R, Cheema T, et al. Guillain-Barré Syndrome associated with SARS-CoV-2 infection. IDCases. 2020;20:e00771.

196. Rajdev K, Victor N, Buckholtz ES, Hariharan P, Saeed MA, Hershberger DM, et al. A Case of Guillain-Barré Syndrome Associated With COVID-19. J Investig Med High Impact Case Rep. 2020 Jan 28;8:232470962096119.

197. Lampe A, Winschel A, Lang C, Steiner T. Guillain-Barré syndrome and SARS-CoV-2. Neurol Res Pract. 2020 Dec 8;2(1):19.

198. Homma Y, Watanabe M, Inoue K, Moritaka T. Coronavirus Disease-19 Pneumonia with Facial Nerve Palsy and Olfactory Disturbance. Internal Medicine. 2020 Jul 15;59(14):1773–5.

199. Avenali M, Martinelli D, Todisco M, Canavero I, Valentino F, Micieli G, et al. Clinical and Electrophysiological Outcome Measures of Patients With Post-Infectious Neurological Syndromes Related to COVID-19 Treated With Intensive Neurorehabilitation. Front Neurol. 2021 Mar 12;12.

200. Egilmez OK, Gündoğan ME, Yılmaz MS, Güven M. Can COVID-19 Cause Peripheral Facial Nerve Palsy? SN Compr Clin Med. 2021 Aug 22;3(8):1707–13.

201. Shouman K, Vanichkachorn G, Cheshire WP, Suarez MD, Shelly S, Lamotte GJ, et al. Autonomic dysfunction following COVID-19 infection: an early experience. Clinical Autonomic Research. 2021 Jun 16;31(3):385–94.

202. Bax F, Lettieri C, Marini A, Pellitteri G, Surcinelli A, Valente M, et al. Clinical and neurophysiological characterization of muscular weakness in severe COVID-19. Neurological Sciences. 2021 Jun 23;42(6):2173–8.

203. Goodman BP, Khoury JA, Blair JE, Grill MF. COVID-19 Dysautonomia. Front Neurol. 2021 Apr 13;12.

204. Guilmot A, Maldonado Slootjes S, Sellimi A, Bronchain M, Hanseeuw B, Belkhir L, et al. Immune-mediated neurological syndromes in SARS-CoV-2-infected patients. J Neurol. 2021 Mar 30;268(3):751–7.

205. Klironomos S, Tzortzakakis A, Kits A, Öhberg C, Kollia E, Ahoromazdae A, et al. Nervous System Involvement in Coronavirus Disease 2019: Results from a Retrospective Consecutive Neuroimaging Cohort. Radiology. 2020 Dec;297(3):E324–34.

206. Garnero M, del Sette M, Assini A, Beronio A, Capello E, Cabona C, et al. COVID-19-related and not related Guillain-Barré syndromes share the same management pitfalls during lock down: The experience of Liguria region in Italy. J Neurol Sci. 2020 Nov;418:117114.
