# Supplementary material for: Peripheral nervous system involvement associated with COVID-19. A systematic review of literature
Source: PLoS One. 2023 Apr 6;18(4):e0283827. doi: 10.1371/journal.pone.0283827 (PMC10079054; doi:10.1371/journal.pone.0283827)
Supplement: Attachment — Submitted filename: Response to Reviewers.docx [file pone.0283827.s003.docx]

Dear editor and reviewers,

Thank you for your valuable comments, allowing us to improve our manuscript.

Please find below the responses to the raised points:

1. Please include captions for your Supporting Information files at the end of your manuscript, and update any in-text citations to match accordingly: - see row 428
2. Please review your reference list to ensure that it is complete and correct.

As suggested, I revised the reference list and there is an article (doi: 10.7759/cureus.14391) that has been retracted because of fake peer review (doi:10.7759/cureus.8992). Thus, we redid the statistics but with no change in the final results. In the original manuscript the article was cited at no. 40. We also eliminated it from the Supplement Table 1.

3. "SARS-CoV-2 is a respiratory virus," - I think here it should be rephrased. SARS-CoV-2 is a respiratory virus spread, but more recent studies show that the primary tropism of SARS-CoV-2 is not limited to the respiratory tract – rephrased, see row 48
4. In the introduction, I recommend deleting the sentence, as since the submission of this manuscript several reviews on this topic have been published: "By contrast, cranial nerve (CN) involvement and other PNS manifestations have not been extensively reviewed." – deleted, see row 60
5. In the methods you state that you have taken into account these data regarding the time of onset. Is there any correlation between specific neurological manifestations and the time of onset? –

The results regarding the duration between disease onset and neurological manifestations were added in table no 4, row 237 and row 386. No statistical association was found.

6. The abstract should be structured according to journal recommendations.

We structured the abstract according to the recommendations: we described the main objective of the study, how the study was performed without details, summarized the most important results and their significance, we did not exceed 300 words and did not use citations.

We used abbreviations (which are not recommended but are not forbidden) because of the frequent use of the terms “cranial nerves” and “peripheral nervous system”. Not using abbreviations would have significantly limited our word count to present our results.

Did we understand correctly that you suggested deleting the subheadings?

7. However, it would be interesting to include a paragraph on the vaccination/ neuropathies relationship in the discussion – see row 282
8. The authors should include some figures to make the text easier to read – see Figure 2, row 242
9. Finally, the bibliography should be expanded; accordingly, this article should be cited: Visco V, Vitale C, Rispoli A, Izzo C, Virtuoso N, Ferruzzi GJ, Santopietro M, Melfi A, Rusciano MR, Maglio A, Di Pietro P, Carrizzo A, Galasso G, Vatrella A, Vecchione C, Ciccarelli M. Post-COVID-19 Syndrome: Involvement and Interactions between Respiratory, Cardiovascular and Nervous Systems. J Clin Med. 2022 Jan 20;11(3):524. doi: 10.3390/jcm11030524. PMID: 35159974; PMCID: PMC8836767 – see reference no 61

In addition, we made some changes at the PRISMA flow chart regarding the number of articles included in the qualitative synthesis because of a transcription error, and in the quantitative analysis because of the retracted paper.

Best wishes,

Andreea Hanganu
